# A Cross-Sectional Observational Study of the Correlation Between Asthma-Related Quality of Life (AQLQ) and Asthma Control Measures

**DOI:** 10.7759/cureus.115818

**Published:** 2026-09-05

**Authors:** Suresh Anantharaj, Ashwini Sathish, Suganya N

**Affiliations:** 1 Respiratory Medicine, Sree Balaji Medical College and Hospital, Chennai, IND; 2 Family Medicine, Sundaram Medical Foundation, Chennai, IND; 3 Epidemiology and Public Health, Sundaram Medical Foundation, Chennai, IND

**Keywords:** acq, aqlq, asthma control, asthma symptoms, gina asthma control, pulmonary function, quality of life

## Abstract

Introduction

The Asthma Control Questionnaire (ACQ) and Asthma Control Test (ACT) are widely used to assess asthma control but are more time-consuming than the Global Initiative for Asthma (GINA) level of control (LoC), a simpler tool that is commonly used in busy clinical settings. Despite the substantial impact of asthma on quality of life, quality-of-life assessment is performed less frequently than assessment of asthma control. This study aimed to evaluate the correlation between the Asthma Quality of Life Questionnaire (AQLQ) and measures of asthma control, including the ACQ and GINA LoC. The primary objective was to compare the strength of the correlations of the ACQ and GINA LoC with the AQLQ.

Methods

Patients with partly controlled or uncontrolled asthma, as defined by the GINA LoC, were recruited after confirmation of the diagnosis by spirometry (post-bronchodilator increase in FEV₁ and/or FVC of >200 mL and >12%) or peak expiratory flow rate (PEFR) criteria (post-bronchodilator improvement in PEFR ≥20%). Baseline demographic and clinical characteristics, including symptoms, smoking status, treatment, and comorbidities, were recorded. All participants completed the AQLQ and ACQ. Correlations of the AQLQ with the ACQ, GINA LoC, and pulmonary function parameters were analyzed. Pearson's correlation coefficient was used to assess the relationships between the AQLQ, pulmonary function parameters (FEV₁ and PEFR), and asthma control measures (ACQ and GINA LoC). Categorical variables, including age distribution and sex, were analyzed using the chi-square test.

Results

Among the 83 patients with asthma, 32 (38.6%) had partly controlled asthma, while 51 (61.4%) had uncontrolled asthma. The mean age and body mass index (BMI) were 43 ± 14.5 years and 28.3 ± 3.9 kg/m², respectively. The proportions of females, patients with more than two symptoms, a positive family history of asthma, allergic rhinitis, and gastroesophageal reflux disease were 47 (56.6%), 17 (20.5%), 28 (33.7%), 17 (20.5%), and nine (10.8%), respectively. The mean ACQ score, AQLQ score, pre-bronchodilator FEV₁ (% predicted), and PEFR (% predicted) were 2.08, 4.4, 58.6%, and 71%, respectively.

The ACQ demonstrated a strong negative correlation with the AQLQ (r = −0.60, p < 0.001), whereas the GINA LoC showed a weak negative correlation with the AQLQ (r = −0.28, p = 0.01). A weak positive correlation was observed between the ACQ and GINA LoC (r = 0.38, p < 0.001). Among pulmonary functions, FEV₁ showed a weak positive correlation with the AQLQ (r = 0.22, p = 0.046) and a weak negative correlation with the GINA LoC (r = −0.24, p = 0.029). PEFR demonstrated a moderate negative correlation with the GINA LoC (r = −0.40, p < 0.001).

Conclusion

Although the ACQ is more time-consuming than the GINA LoC, it demonstrated a stronger correlation with asthma-specific quality of life and may be a better predictor of quality of life in adults with asthma.

## Introduction

Asthma is a common chronic respiratory disease affecting people of all ages worldwide and significantly impacting quality of life. Approximately 300 million people globally have asthma [1]. In India, a large-scale analysis of the 2017-2018 National Sample Survey reported an overall asthma prevalence of 2.0 per 1000 population, with a prevalence of 1.9 per 1000 in rural areas and 2.3 per 1000 in urban areas. The study also demonstrated significant associations between asthma prevalence and sociodemographic and household factors, including age, sex, educational status, place of residence, and cooking fuel [2].

Earlier asthma management guidelines primarily focused on minimizing symptoms, optimizing lung function, and preventing exacerbations, with lung function often considered the primary outcome. However, studies later demonstrated a poor correlation between lung function and clinical symptoms. Consequently, both clinical practice and research shifted their focus toward the broader concept of "asthma control." The Global Initiative for Asthma (GINA) guidelines define asthma control based on two domains: symptom control and future risk. This comprehensive assessment includes symptoms, reliever medication use, lung function, and the frequency and severity of exacerbations [1]. Although several tools are available to assess asthma control, numerical instruments such as the Asthma Control Questionnaire (ACQ) and Asthma Control Test (ACT) are more sensitive to changes in symptom control than categorical measures and are widely used in clinical research [3-5].

Despite their usefulness, asthma control measures do not necessarily reflect the impact of asthma on patients' quality of life. Achieving good symptom control and improved lung function does not always translate into better quality of life. Patients may experience relief from symptoms while continuing to face emotional, environmental, or activity-related limitations that affect their overall well-being.

Recognizing the importance of patient-centered outcomes, the concept of quality of life was incorporated into the GINA guidelines in 2009. The Asthma Quality of Life Questionnaire (AQLQ), developed and validated by Juniper, assesses quality of life across four domains: symptoms, activity limitation, emotional function, and environmental exposure. Two versions are available: the standard AQLQ, consisting of 32 items that comprehensively assess the daily challenges faced by patients with asthma, and the Mini-AQLQ, a shorter 15-item version designed for easier administration [6,7].

Although the AQLQ is a validated instrument, administering a lengthy questionnaire in busy outpatient settings is often impractical, particularly in resource-limited countries such as India. Therefore, clinicians frequently rely on simpler asthma control tools as surrogate measures of quality of life. However, whether these tools accurately reflect the quality-of-life impairment experienced by patients remains uncertain, as they primarily assess disease control rather than broader patient well-being.

The AQLQ has demonstrated acceptable validity, reliability, and discriminatory properties among Indian patients with asthma and is considered an appropriate instrument for assessing quality of life in both clinical practice and research. Previous studies have reported significant associations between asthma control and quality of life. Siroux et al. reported progressively lower AQLQ scores across controlled, partly controlled, and uncontrolled asthma categories based on asthma control criteria adapted from the GINA guidelines [8]. Similarly, Sadatsafavi et al. demonstrated a significant association between symptom control and disease-specific quality of life [9]. More specifically, Pizzichini et al. evaluated the relationship between the current GINA definition of asthma control and AQLQ and found significantly better quality of life among patients with controlled asthma compared with those with partly controlled or uncontrolled asthma [10]. However, studies specifically evaluating the strength of the relationship between GINA level of control (LoC) and AQLQ remain limited.

Therefore, this study aimed to evaluate the relationship between AQLQ and measures of asthma control in adults with partly controlled or uncontrolled asthma. The primary objective was to compare the strength of the correlation between AQLQ scores and the ACQ with that between AQLQ scores and the GINA LoC. The secondary objectives were to assess the correlation of AQLQ scores with pulmonary function parameters, including forced expiratory volume in one second (FEV₁) and peak expiratory flow rate (PEFR), and to evaluate the association between asthma control categories and asthma-related quality of life.

## Materials and methods

Study design and study population

This was a cross-sectional observational study. Ethical approval was obtained from the Institutional Review Board of Sundaram Medical Foundation (Ref No: 108-41186-122-105688). Informed consent was obtained from all participants before data collection.

Using the formula, the sample size was calculated: N = 2σ^2^(Z_β_​+Z_α/2_​)^2^/D^2^​​​​​​. On substituting the values, σ = 1.04; Z_β_ = 0.84; Z_α/2_ = 1.96; D = 0.452; α = 5%; and power = 80%. Hence, the final sample size was 83 participants. The inclusion and exclusion criteria for the study are summarized in Table 1.

**Table 1:** Inclusion and exclusion criteria

Category	Criteria
Inclusion criteria	Adults aged 18-65 years
	Patients with partly controlled or uncontrolled asthma, as defined by the Global Initiative for Asthma (GINA) level of control (LoC)
	Patients who had not received inhaler therapy previously
Exclusion criteria	Acute asthma exacerbation
	Chronic rhinosinusitis
	Bronchiectasis
	Chronic obstructive pulmonary disease (COPD)
	Newly diagnosed pulmonary tuberculosis
	Other chronic respiratory illnesses
	Left ventricular (LV) dysfunction
	Chronic kidney disease (CKD) stage IV or V

Methodology

The study was conducted at Sundaram Medical Foundation, Dr. Rangarajan Memorial Hospital, over a period of two years. Both newly diagnosed and previously diagnosed patients with asthma, as defined by the GINA guidelines, were eligible for inclusion if they had previously received inhaler therapy and had partly controlled or uncontrolled asthma according to the GINA LoC. Patients who had not received inhaler therapy previously were excluded (Figure 1). Basic demographic and clinical characteristics, such as age, BMI, symptoms, details about comorbidities such as allergic rhinitis, gastroesophageal reflux disorder, smoking status, family history, and treatment details, were recorded.

**Figure 1:**
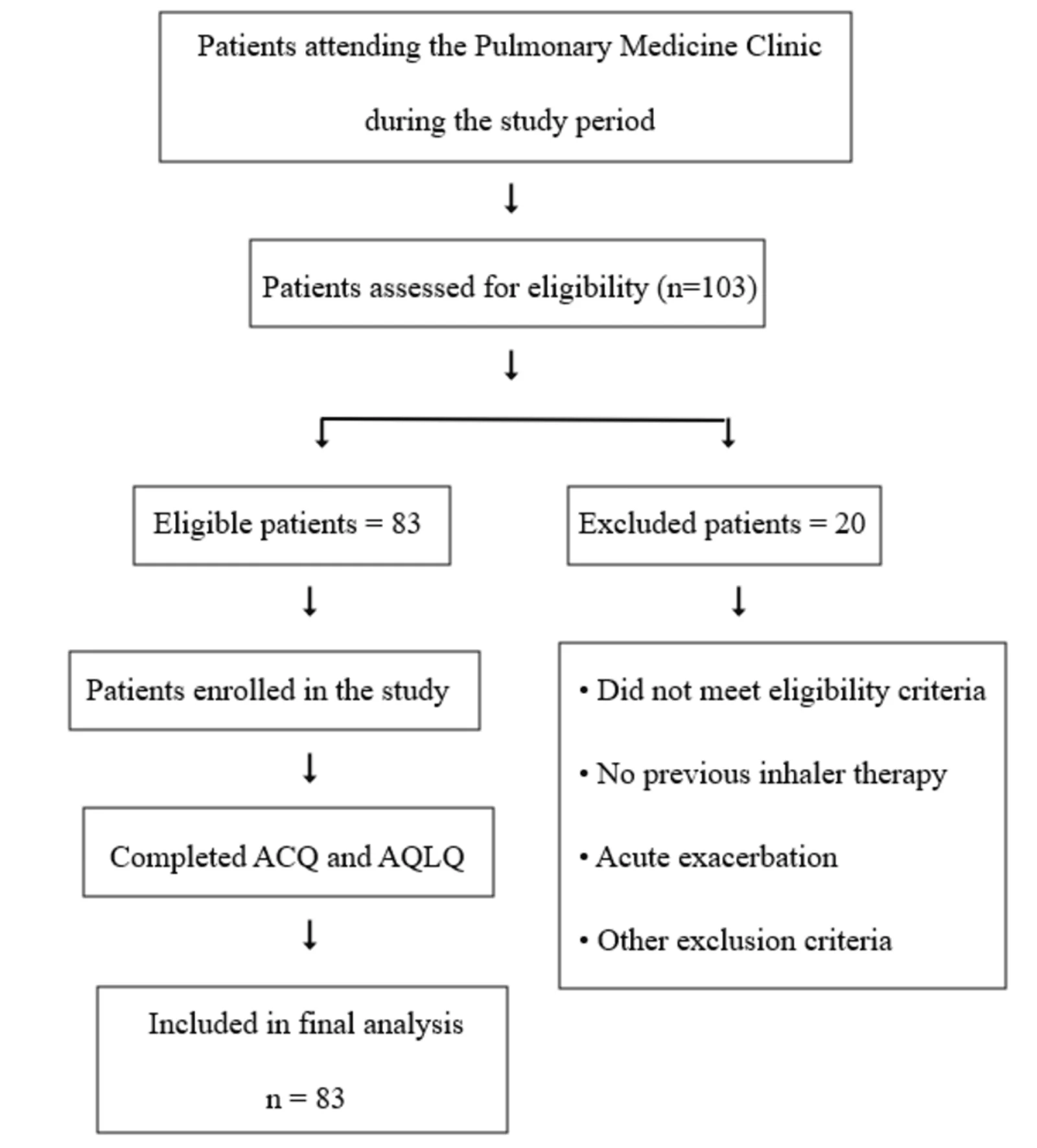
STROBE flowchart STROBE: Strengthening the Reporting of Observational Studies in Epidemiology; ACQ: Asthma Control Questionnaire; AQLQ: Asthma Quality of Life Questionnaire Source: [11]

Asthma was diagnosed based on characteristic clinical symptoms and demonstration of significant post-bronchodilator reversibility on spirometry using a Ganshorn spirometer. Reversibility was defined as a significant improvement in FEV₁ and/or forced vital capacity (FVC). Patients demonstrating a post-bronchodilator improvement in peak expiratory flow (PEF) of ≥20% from the pre-bronchodilator value were also included [1]. Scoring of AQLQ and ACQ was done. Correlation between these parameters was analyzed statistically. 

Statistical analysis

The AQLQ and ACQ scores were expressed as mean ± standard deviation (SD). Pearson's correlation coefficient was used to assess the relationship between AQLQ, pulmonary function parameters (FEV₁ and PEFR), and asthma control measures (ACQ and GINA LoC).

The AQLQ scores were categorized into intervals of 0-1, 1-2, …, and 6-7, with higher scores indicating a better quality of life and lower scores indicating a poorer quality of life. An ACQ score of ≥1.5 was considered uncontrolled asthma, while a score of <1.5 was considered partly controlled or well controlled (0 = <1.5; 1 = ≥1.5). FEV₁ and PEFR values <80% of the predicted value were classified as decreased (0 = <80%; 1 = ≥80%). The GINA LoC was categorized as 0 = partly controlled and 1 = uncontrolled. Symptom burden was categorized as <2 symptoms and ≥2 symptoms.

Data were entered into MS Excel (Microsoft Corporation, Redmond, Washington, United States) and analyzed using the IBM SPSS Statistics for Windows, Version 19 (Released 2010; IBM Corp., Armonk, New York, United States). Categorical variables, including age distribution and sex, were analyzed using the chi-square test. A two-tailed p-value of ≤0.05 was considered statistically significant for all analyses.

## Results

A total of 83 adult patients with asthma were included in the study. According to the GINA LoC assessment, 32 (38.6%) patients had partly controlled asthma, while the remaining 51 (61.4%) had uncontrolled asthma. More than half of the patients (n = 45, 55%) were older than 40 years, with a female predominance (n = 47, 56.6%). Over half of the study population (n = 43, 51.8%) had a BMI above the upper limit of normal (>24.9 kg/m²). Seventeen of the 83 patients reported more than two asthma-related symptoms, and 28 (33.73%) had a family history of asthma. The prevalence of asthma-associated comorbidities was 17 (20.4%) for allergic rhinitis, nine (10.8%) for gastroesophageal reflux disease, and nine (10.8%) for smoking. Comparison of baseline demographic and clinical characteristics between the partly controlled and uncontrolled groups showed a significantly higher proportion of females and smokers in the uncontrolled group (Tables 2-3).

**Table 2:** Baseline and clinical characteristics comparison between groups p-value < 0.05: significant; NS: nonsignificant

Category	All (mean/proportion)	Partly controlled	Uncontrolled	p-value
Age	43.0 ± 14.5	44.4 ± 14.0	41.9 ± 15	(NS)
Gender (N = 83)				
Male	N = 36 (43.4%)	17 (47.2%)	19 (52.8%)	(NS)
Female	N = 47 (56.6%)	15 (31.9%)	32 (68.1%)	<0.05
BMI	28.3 ± 3.9	25.5 ± 5.7	30 ± 9.2	(NS)
Symptoms (>2)	N = 17 (20.5%)	N = 10 (58.8%)	N = 7 (41.2%)	.064 (NS)
Allergic rhinitis	N = 17 (20.5%)	N = 7 (41.2%)	N = 10 (58.8%)	(NS)
Gastroesophageal reflux	N = 9 (10.8%)	N = 4 (44.4%)	N = 5 (55.6%)	(NS)
Family history	N = 28 (33.73%)	N = 13 (46.4%)	N = 15(53.6%)	(NS)
Smoking	N = 9 (10.8%)	N = 3 (33.3%)	N = 6 (66.7%)	<0.05

**Table 3:** Baseline and clinical characteristics-statistical comparison between groups χ²: Chi-square value; df: degree of freedom

Variable	χ²	df	p-value	Effect size (φ)
Gender	2.016	1	0.156	0.156
Symptoms >2	3.708	1	0.064	0.211
Allergic rhinitis	0.062	1	0.803	0.027
Gastroesophageal reflux	0.148	1	0.701	0.042
Family history	1.106	1	0.293	0.115
Smoking	0.116	1	0.733	0.037

Among the 83 patients, asthma was diagnosed by bronchodilator reversibility on spirometry (FEV₁ and/or FVC) in 64 (77.1%) patients, by reversibility in PEFR in 12 (14.5%) patients, and by both methods in seven (8.4%) patients. The mean prebronchodilator PEFR and FEV₁ were 71.02% and 58.58% of the predicted values, respectively. The mean ACQ and AQLQ scores for the study population were 2.08 and 4.4, respectively. Comparison of the mean ACQ and AQLQ scores and pulmonary function parameters between the partly controlled and uncontrolled groups showed no statistically significant differences. However, the differences in the mean ACQ and AQLQ scores between the two groups were 1.1 and 0.7, respectively, both exceeding the minimal clinically important difference (MCID) of 0.5. This suggests that, despite the absence of statistical significance, the uncontrolled group had clinically meaningful impairment in asthma control and quality of life (Table 4).

**Table 4:** Asthma control, quality of life, and pulmonary function comparison between groups ACQ: Asthma Control Questionnaire; AQLQ: Asthma Quality of Life Questionnaire; PEFR: peak expiratory flow rate; NS: nonsignificant; p-value < 0.05: significant

Category	All (mean)	Partly controlled (mean)	Uncontrolled (mean)	p-value
ACQ	2.08	1.4	2.5	0.094 (NS)
AQLQ	4.4	4.8	4.1	0.614 (NS)
FEV1 (% predicted)	58.6	62.5	56.2	0.754 (NS)
PEFR (% predicted)	71	79.7	65.6	0.429 (NS)

The ACQ demonstrated a strong negative correlation with the AQLQ (r = −0.60, p < 0.001) (Figure 2), whereas the GINA LoC showed a weak negative correlation with the AQLQ (r = −0.28, p = 0.01). A weak positive correlation was observed between the ACQ and GINA LoC (r = 0.38, p < 0.001).

**Figure 2:**
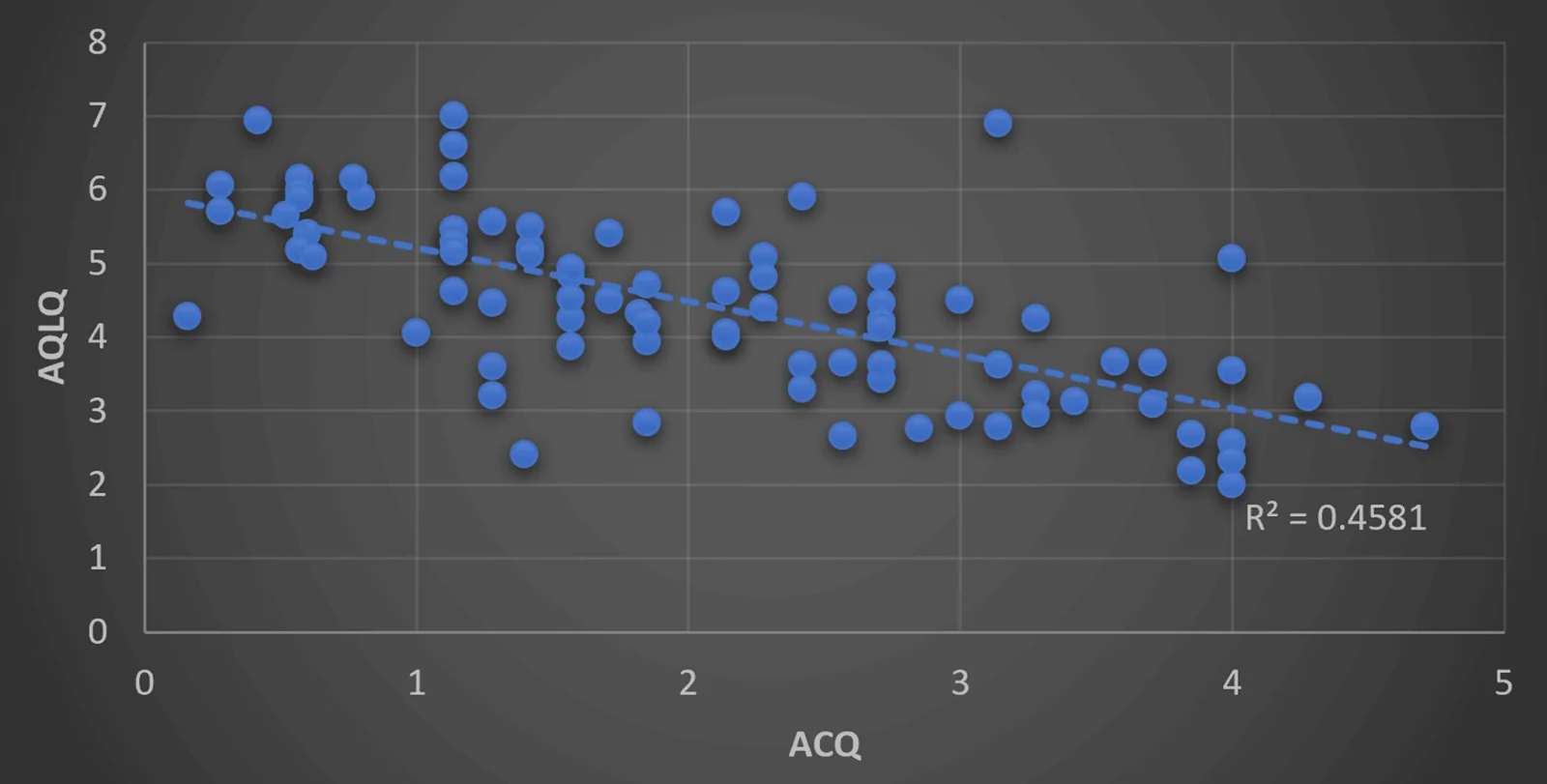
AQLQ vs ACQ ACQ: Asthma Control Questionnaire; AQLQ: Asthma Quality of Life Questionnaire

Among the pulmonary function parameters, FEV₁ showed a weak positive correlation with the AQLQ (r = 0.22, p = 0.046) (Figure 3) and a weak negative correlation with the GINA LoC (r = −0.24, p = 0.029). PEFR demonstrated a moderate negative correlation with the GINA LoC (r = −0.40, p < 0.001).

**Figure 3:**
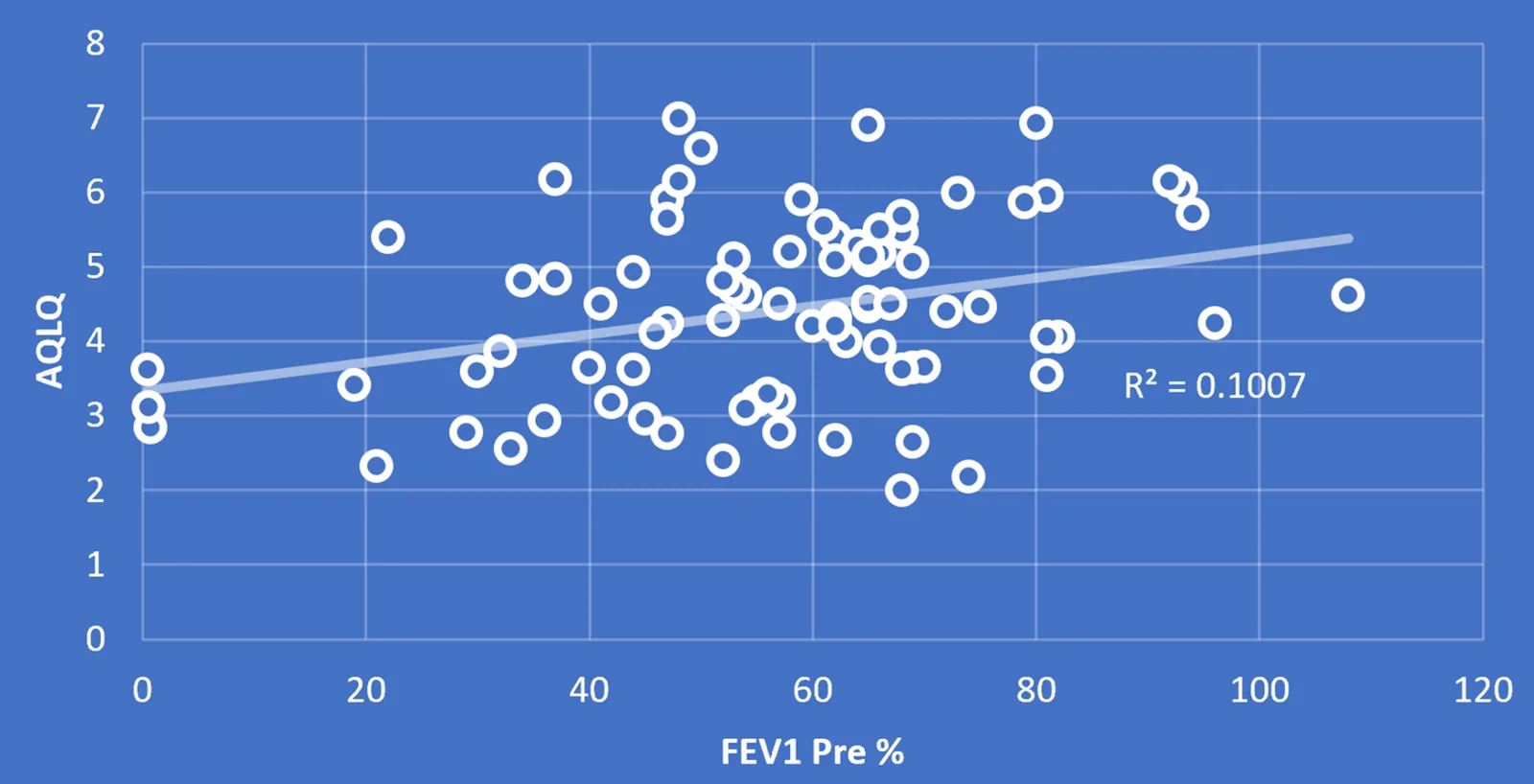
AQLQ vs FEV1 AQLQ: Asthma Quality of Life Questionnaire; FEV1: forced expiratory volume in 1 second

No significant correlation was observed between either pulmonary function parameter and the ACQ or between PEFR and the AQLQ (Figure 4).

**Figure 4:**
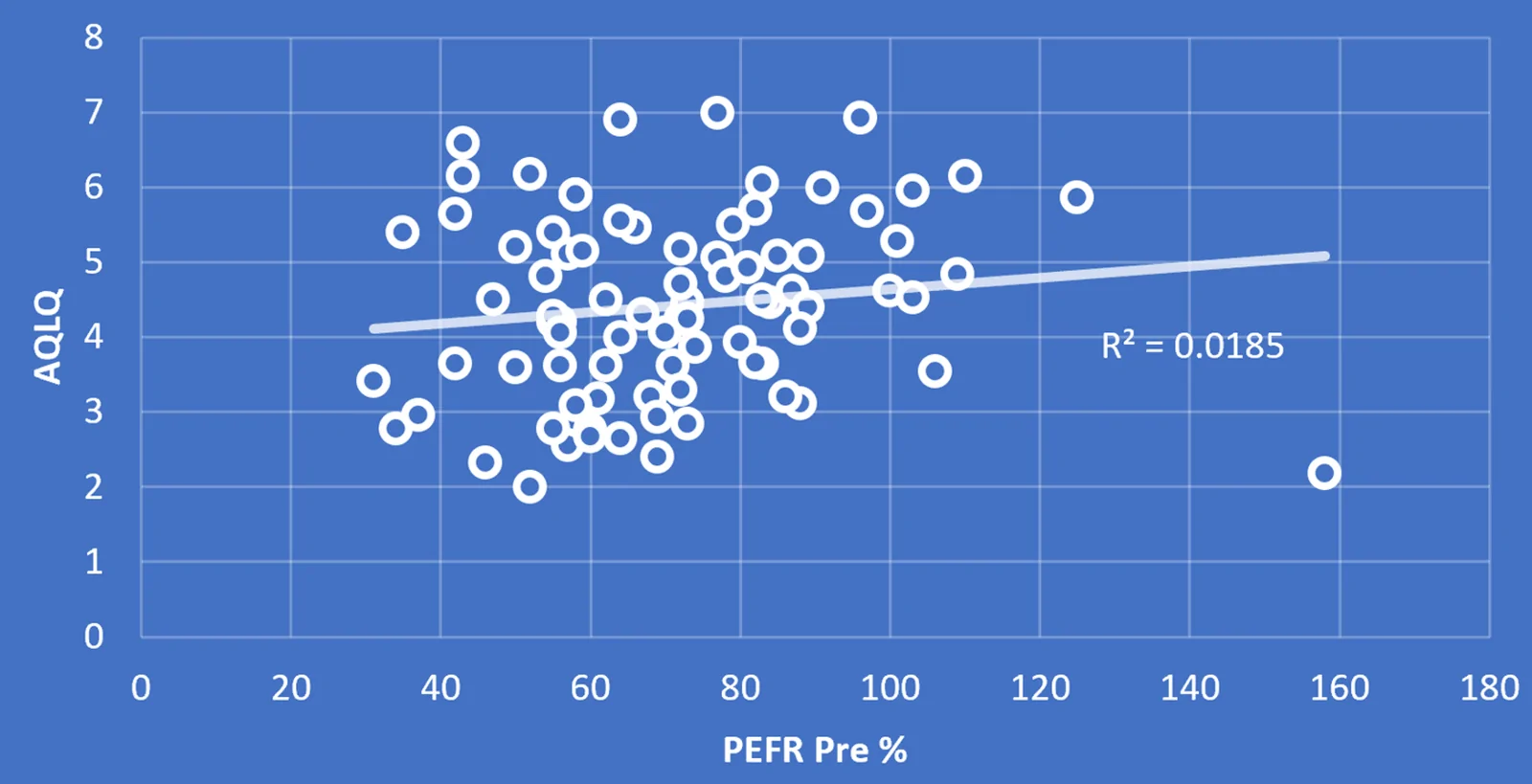
AQLQ vs PEFR AQLQ: Asthma Quality of Life Questionnaire; PEFR: peak expiratory flow rate

## Discussion

Significant associations were observed between some asthma control measures and asthma-related quality of life. Among the asthma control measures, the ACQ, a quantitative measure of asthma control, showed a strong negative correlation with asthma-related quality of life. Previous studies have compared the AQLQ with asthma control measures such as the ACQ and the ACT [12]. Our findings are consistent with those of similar cross-sectional studies, including those by Braido et al., demonstrating a significant association between ACQ scores and AQLQ [13].

In a study by Siroux et al. [8], the ACQ was compared with the AQLQ, and the cross-sectional analysis demonstrated a significant, strong negative correlation (r = −0.62, p < 0.01). This inverse relationship is expected because higher ACQ scores indicate poorer asthma control, whereas higher AQLQ scores indicate better quality of life; therefore, worsening asthma control is associated with poorer quality of life. In the same study, the longitudinal correlation between changes in ACQ scores and changes in AQLQ scores was moderate but statistically significant (r = 0.58, p < 0.01). Similarly, Correia de Sousa et al. [14] reported a strong positive correlation between asthma control, measured using the ACT, and quality of life, measured using the Mini-Asthma Quality of Life Questionnaire (Mini-AQLQ) (r = 0.81, p < 0.001). The positive correlation is because higher ACT scores indicate better asthma control, while higher Mini-AQLQ scores indicate better quality of life. Thus, the direction of the correlation depends on how each asthma control instrument is scored, while the overall findings indicate that better asthma control is associated with better asthma-related quality of life.

Although studies from other centres have reported good correlations between GINA LoC and quality of life, our study demonstrated only a weak negative correlation between GINA LoC and AQLQ scores. A weak positive correlation was observed between the two asthma control measures, ACQ and GINA LoC (r = 0.38, p < 0.001), indicating that the two measures were associated but did not demonstrate a strong concordance. This difference may partly reflect the categorical nature of the GINA LoC assessment compared with the numerical scoring of the ACQ, which may allow the ACQ to capture gradations in asthma control that may not be reflected by a categorical classification. Despite the absence of statistical significance in the comparison of AQLQ scores between the partly controlled and uncontrolled groups, the difference exceeded the minimally clinically important difference (MCID) of 0.5, suggesting a clinically meaningful difference in quality of life. The stronger observed correlation between ACQ and AQLQ suggests that ACQ may have a closer association with asthma-related quality of life in this study population; however, a formal statistical comparison of the correlation coefficients would be required to establish whether this difference is statistically significant.

Regarding pulmonary function parameters, FEV₁ showed a weak positive correlation with AQLQ (r = 0.22, p = 0.046), indicating that better lung function was associated with better asthma-related quality of life, although the association was weak. FEV₁ also showed a weak negative correlation with GINA LoC (r = −0.24, p = 0.029), while PEFR demonstrated a moderate negative correlation with GINA LoC (r = −0.40, p < 0.001). In contrast, PEFR did not show a significant correlation with AQLQ, and neither FEV₁ nor PEFR demonstrated a significant correlation with ACQ. These findings suggest that pulmonary function parameters may show some association with categorical asthma control but may not consistently correspond with patient-reported asthma control or quality of life.

Although the ACQ and ACT have consistently been shown to be associated with asthma-related quality of life, their use may be less practical in busy outpatient settings. The GINA LoC assessment is simpler, relying on only four questions addressing daytime symptoms, nocturnal symptoms, activity limitation, and reliever medication use. This makes it a practical tool in a highly populated country such as India, where healthcare settings are often busy, and literacy levels vary considerably. Previous studies have demonstrated significant associations between GINA LoC and disease-specific quality-of-life measures such as the AQLQ and AQ5D, as well as generic instruments including the SF-36 and EQ-5D [8,9]. However, differences in the study populations and clinical settings limit the generalizability of these findings to our population.

Limitations

This was a single-center study, and the sample size was insufficient to generalize the findings to the broader population. As this was a cross-sectional study, the observed associations between asthma control measures and asthma-related quality of life cannot establish a temporal relationship, causality, or predictive ability. In addition, our study population had varying abilities to complete self-administered questionnaires, such as the ACQ and the AQLQ, which may have influenced the results to a small extent. Larger, multicenter studies with more diverse populations and longitudinal designs are needed to address these limitations and further validate our findings.

## Conclusions

Assessment of AQLQ is a difficult and time-consuming process in busy clinical settings. Although the GINA LoC assessment provides a simplified approach to assessing asthma control, it demonstrated only a weak correlation with AQLQ in our study. The ACQ demonstrated a stronger observed correlation with AQLQ than the GINA LoC assessment. However, a formal statistical comparison between the correlation coefficients was not performed; these findings should not be interpreted as demonstrating superiority of ACQ over GINA LoC. Further studies with larger sample sizes are required to determine whether ACQ provides a significantly stronger association with asthma-related quality of life.
